# Ecological correlates related to adolescent movement behaviors: A latent class analysis

**DOI:** 10.1371/journal.pone.0271111

**Published:** 2022-07-21

**Authors:** Isabella Toledo Caetano, Valter Paulo Neves Miranda, Fernanda Karina dos Santos, Paulo Roberto dos Santos Amorim

**Affiliations:** Department of Physical Education, Federal University of Viçosa, Viçosa, Minas Gerais, Brazil; International Center for Theoretical Physics - South American Institute for Fundamental Research, BRAZIL

## Abstract

The ecological model has been widely used to help researchers understand the multiple influences in the physical activity (PA) and in the sedentary behaviors in isolated forms. To date, few correlates concerning the behavioral groupings of PA and sedentary behaviors have been studied. In this context, this study aimed to identify movement behaviors’ latent classes related to the different adolescents’ PA and sedentary time expressions, as well as their associations with individual, sociodemographic, family, and environmental correlates. This is a cross-sectional study with 309 students aged between 14 and 16. Latent Class Analysis was used to identify movement behavior classes based on light PA, moderate to vigorous PA, number of steps, sedentary time, and screen time (ST). An accelerometer was used to evaluate movement behaviors. The individual, sociodemographic, family, and environmental correlates were assessed by questionnaires. Three classes were identified: Class 1, "Active and Non-Sedentary" (8.10% of the sample), Class 2, "Active and Sedentary" (28.5%), and Class 3, "Inactive and Sedentary" (63.4%). Those with low fruit intake, low aerobic fitness, stressed and whose head of the family obtained an ‘elementary school’ level education were, respectively, 7.17, 3.59, 3.56, and 4.40 times more likely to belong to class 3 than class 1. Those with medium and high socioeconomic status were 82% and 83% less likely to belong to class 1 than classes 2 and 3, respectively. Adolescents who perceived the neighborhoods with the best access to diversified land use, street connectivity, walking/pedaling ease, and traffic safety attributes, were 84%, 85%, 82%, and 82%, respectively less likely to belong to class 1 than class 2. It is concluded that distinct correlates can be associated with the movement behaviors classes.

## Introduction

Physical activity (PA) and sedentary behavior are considered movement behaviors [1] associated with lifestyle; reduced PA levels and high sedentary time are recognized as significant risk factors for the development of chronic diseases in adolescence [2]. These movement behavior patterns have increasingly affected the pediatric population. Recent global statistics show that more than three-quarters (81%) of adolescents do not meet the global PA recommendations [3]. In addition, national data from the Study of Cardiovascular Risks in Adolescents (ERICA) indicate that approximately 56% of Brazilian adolescents aged between 14 and 17 years do not reach 300 minutes of PA per week [4]. ERICA also showed that 73.8% of more than 40.000 adolescents aged between 15 and 17 reported spending two or more hours a day in front of the television, computer, or video games [5].

In addition, the evidence indicates a high sedentary time, and engagement in specific sedentary activities as television viewing time or recreational screen time (ST) that have rapidly emerged as potential additional risk factors for adolescents’ health [6]. Nonetheless, the ways in which adolescents interact with screens have also rapidly changed. For example, although television viewing was the main source of US adolescents’ screen time in the late-1970s, in 2016, it consisted of only 25% of their overall screen use [7]. Emerging evidence points to an increase in the use of technologies and mobile internet, such as desktop computers, cell phones, laptops, and other types of devices such as iPod or mp3 among young people [8–10], as well as the association of this behavior with negative health outcomes [11].

Although many studies have suggested low PA and high sedentary time, this is not the case for all adolescents [12], as adolescents may be involved in different PA related behavior combinations [13–15]. For example, there are groups of adolescents who engage in good levels of a particular behavior combined with harmful levels of another behavior, as well as a combination of favorable or harmful levels of both behaviors [13, 14].

Latent Class Analysis (LCA) is a robust and multivariate statistical method, that has been more recently used in epidemiological studies to identify behavioral patterns [16]. It is an appropriate cluster analysis technique to identify groups or PA and sedentary behavior classes related to health behaviors [13, 17, 18]. In addition, it analyses iterations and associations between different types of behavioral variables related to the sample’s specific characteristics [19].

In this context, a variety of researches seeks to understand the factors that influence individual behaviors related to PA or sedentary behavior [12, 20, 21]. However, only a few studies seek to understand the primary factors that can influence adolescents’ movement behavior combinations.

Although PA and sedentary behavior are two different behavioral constructs [22, 23], it has been suggested that their correlates may be similar [22, 23]. Because both arise from intrapersonal characteristics (biological, behavioral, psychological, and demographic correlates) [24–26], interpersonal (social and cultural correlates) [27], environmental (social and physical correlates–build and natural) [28, 29], organizational and political [25, 26]. Thus, their individual expressions are the result of a complex interaction of behavioral, biological, and environmental factors [22, 23, 30].

A theoretical model that seeks to understand human interactions is the ecological model. It places the individual at the center of an ecosystem and provides a better understanding of the various factors that impact a given behavior [30], which suggests that a combination of factors at multiple levels (intrapersonal, interpersonal, environmental) interact to influence an individual’s behavior [31]. Some correlates have been associated with the groupings of behaviors related to PA and sedentary time in children and adolescents, being the most studied the sociodemographic (SS and schooling) [12, 13] and individual (gender, age, and BMI) correlates [13, 17, 18, 32].

In the literature, we only identified one study [13] that sought to understand several factors (intrapersonal, interpersonal, and physical) that inhibit or facilitate movement behaviors combinations engagement in young people from Melbourne, Australia. Although this study is consistent in its findings, it is known that the context in which the individual is inserted can have a great influence on their behavior. Thus, it is also interesting to investigate a set of ecological correlations between PA classes and sedentary time of adolescents from a small town in Brazil, a developing country, since the evidence in the literature is predominantly from Western Europe, North America, and Australia.

Identifying these correlates can contribute to a better understanding of the main factors that influence the behavior configurations related to adolescents’ activity, as well as assist in structuring strategies focused on the individual, family, sociodemographic, and neighborhood constructed environment domains. Thus, this study aimed to identify movement behaviors latent classes related to the different adolescents’ PA and sedentary time expressions, as well as their associations with individual, sociodemographic, family, and environmental correlates.

## Materials and methods

### Study design and participants

This cross-sectional study was conducted between March and December 2019, with adolescents of both sexes, aged between 14 and 16 years, regularly enrolled in the first year of high school in the public schools (five state schools and one federal) of the city of Viçosa, Minas Gerais, Brazil.

The study protocol was conducted according to the Declaration of Helsinki principles and approved by the Research Ethics Committee involving human beings of the Federal University of Viçosa, under the decision number 00925118.6.0000.5153. Before taking any action, participants and their parents or legal guardians signed the Free and Informed Consent (FIC) and the Assent (FIA) forms.

The sample size was determined using a specific formula in the EpiInfo software, version 7.2.2.16, for cross-sectional studies (Georgia, USA). The defined population size was 968 (total number of students enrolled in the first year of high school in the city’s public schools), and the results prevalence is 50% since the study considers multiple health behaviors of the adolescent population [33]. A 5% acceptable error, a 95% confidence level, and a 1.1 drawing effect were adopted. In these configurations, a minimum sample size of 305 adolescents was found. 20% were added to this calculation to recover possible losses. Six schools were selected to participete. In each of the 6 schools was carried out a draw at random, based on the list of enrolled students [34], to select the students who would participate in the study. The draw was carried out so that all students from participating schools had the same chance to participate. Data were collected from 367 adolescents.

To be included in the study, adolescents should be aged between 14 and 16 years, return signed FIC and FIA forms, and be regularly enrolled in the first year of high school. Exclusion criteria included pregnancy and temporary or permanent physical or mental disability.

A team of previously trained evaluators carried out the evaluations. The collection occurred in four meetings with each participant. In the first meeting, the adolescents received information related to the research and its procedures, were officially invited to participate, and received the FIC and FIA forms. In the second meeting, which lasted on average 60 minutes, the adolescents delivered the signed forms and filled out the research questionnaires, in the classroom, with the help of the study’s first author. In the third meeting, held in the school’s sports courts, lasting approximately 30 minutes, the cardiorespiratory fitness test was performed, followed by the accelerometer placement, for the direct PA and sedentary time evaluation. A verbal explanation of the use of the device was provided along with an equivalent instructions sheet, and a daily use journal was also provided that should be filled in when the monitor was removed from the body and during night sleep times. In the same meeting, each student received the questionnaires to take home and these should be answered with information regarding the socioeconomic level of the family and the head of the family’s PA behaviors. In the fourth meeting, students should return with the questionnaire filled out and deliver the accelerometer along with the daily use journal.

#### Movement behaviors evaluation

*Physical activity*, *number of steps*, *and sedentary behavior–Accelerometer*. The ActiGraph accelerometer (GT3X model) was used to measure light PA (LPA), moderate to vigorous PA (MVPA), number of steps, and sedentary time. The ActiLife software (version 6.13.4) (ActiGraph, LLC, Fort Walton Beach, USA) was used to perform all accelerometer analyses. The adolescents used the monitors on the right hip on an elastic belt for eight consecutive days, including during night sleep. The adolescents were instructed not to change their daily routine, and the accelerometer should only be removed for aquatic activities, such as bathing and swimming. Each evaluated person received an equipment use daily journal, in which they should write down the time they woke up and slept every day, and the moments when the monitor was removed from the body and replaced. The first day of use (the day they received the device) was not considered in the analysis to avoid the Hawthorne Effect [35].

The accelerometer was initialized to collect data at a sampling rate of 30Hz, with a normal filter, and the data was reintegrated into 15s epochs. The non-use time was defined as zero consecutive counts/minute for at least 20 minutes. To be included in the analysis, the participants needed to reach a minimum of 10 h.day^-1^ of "time of use" [36], and at least five days a week, of which at least one day should be a weekend day. Sleep time analyzes were performed using the accelerometer use diary (time I went to sleep / time I woke up) filled in by the adolescents. To aid in these analyses, accelerometer information containing daily graphs, inclinometer data, and comma-separated values (.csv) files were converted into a data table to calculate average sleep duration. These sleep/wake times were used to create the subjects’ record journals and removed from the analysis. We adopted the cut-off points developed by Romanzini et al. [37] validated for Brazilian adolescents, using magnitude vector and 15s epochs to classify PA and sedentary time.

*Screen time—Self-report*. The total screen time (ST) was evaluated by the "Portable Technologies and Mobile Internet Questionnaire" (TECNOq) [38]. A score in minutes was created for each type of portable technology (mobile phone, tablet, and notebook) evaluated. To verify the total ST, the scores in minutes of the three technologies were added.

### Ecological correlates

In the present study, biological characteristics, lifestyle habits (referring to fruit intake, commuting mode, work, and cardiorespiratory fitness) and psychological characteristics were considered Individual Correlates. The head of the family’s schooling and family income were deemed to be Sociodemographic Correlates. The PA level of the head of the family was considered Family Correlate. The neighborhood’s built environment characteristics were considered Environmental Correlates. Following, each correlate will be approached individually, and its features described.

### Individual correlates

#### Biological and psychological characteristics and lifestyle habits

Biological characteristics such as age and sex; lifestyle habits related to alcohol, fruits, vegetables and sugars intake, work, and commuting mode to school, as well as psychological characteristics such as stress level, feeling of loneliness and sadness were evaluated by specific questions of the "Risk Behaviors of Adolescents from Santa Catarina—COMPAC" questionnaire [39].

Regarding the weekly frequency of fruits, vegetables, and sugars intake, it was considered "high intake" for consumptions that were equal to or greater than three days a week for each food group. When considering alcohol intake, those who responded never having consumed alcohol were categorized as "does not consume alcohol" and those who consumed at least one dose per week, or more were categorized as part of the "consumes alcohol" group.

Regarding work, adolescents answered the question: “Do you work?” Response options were: 0 = no working; 1 = yes, I work up to 20 hours/week; 2 = yes, I work more than 20 hours/week. So, adolescents who work 20 hours/week or more were placed in the "yes, I work" category, and the others in the "I do not work" category. The travel mode was recategorized into "active commuting" for those who commute on foot or by bike to school and "passive commuting" for those who use motorcycles, cars, or buses. For the feeling of loneliness, the answers were recategorized as "never lonely" and "lonely"; for the feeling of sadness, the answers were categorized as "yes sadness" or "no sadness", finally, for the stress level, responses were recategorized into "no stress" and "stressed" groups.

#### Cardiorespiratory fitness

Cardiorespiratory fitness (CRF) was evaluated through a 20m Shuttle Run running test [40]. The 50th percentile (50^th^ P) was applied to the data set to classify the CRF; thus, when the CRF was above 50^th^ P, it was considered an "adequate" cardiorespiratory capacity.

### Sociodemographic correlates

The SS was classified through a specific questionnaire suggested by the Brazilian Association of Research Companies [41], which was answered by the head of the family. A particular questionnaire question made it possible to evaluate the head of the family’s level of education, whose answers were recategorized as "never studied", "elementary school", "high school", and "higher education".

### Family correlates

The head of the family’s PA level was evaluated by the short version of the International Physical Activity Questionnaire (IPAQ) [42], previously validated for Brazilian adults. PA was evaluated through the application of sections 1 to 3. The questionnaire considers the frequency, duration and intensity of activities and its classification is based on scores obtained through the sum of time and days involved in walking and activities of moderate intensity and vigorous. Self-reported values were recorded and mean PA values per week were obtained. For the present study, only the information referring to MVPA was used, where the internationally accepted cut-off point of at least 150 minutes of MVPA per week was adopted [43]. Thus, individuals were considered "MVPA active" when the MVPA was ≥ 150 minutes per week.

### Environmental correlates

The neighborhood’s built environment characteristics were evaluated by the Neighborhood Walkability for Youth Scale (NEWS-Y) [44]. NEWS-Y assesses eight domains regarding the adolescents’ perception of diversified land use, residential density, access to diversified land use, street connectivity, walking and cycling ease, pedestrian and car traffic safety, crime safety and neighborhood aesthetics. An average was calculated for each of the domains, so that values greater than the average indicated higher values of the respective domain [44, 45].

### Latent class manifest variables

Five manifest variables related to the different PA and the sedentary time expressions were selected to describe the adolescent’s movement behaviors classes: LPA, MVPA, number of steps, sedentary time, and ST.

The variables were categorized dichotomously to facilitate results interpretation. The MVPA was considered adequate when the participants performed 60 minutes per day [46]. Unfortunately, there are still no specific cut-off points for LPA and total sedentary time. Therefore, we chose to work with percentile values. Thus, the 25th percentile (25thP), and the 75th percentile (75thP) were used as cutoff points because they were the values that resulted in a better fit of the variables observed in the latent variable model. Furthermore, they were the same cutoff points used by Faria et al. [17] and Caetano et al. [47]. The 75thP was also applied to the number of steps, as previously used by Faria et al. [17] and Miranda et al. [18] since only a small percentage of participants (13.3%) reached the cut-off point of 11,700 steps proposed by Tudor-Locke et al. [48]. The 25thP was also applied to ST since only a small percentage of participants (8.4%) were below the cut-off point of 2 hours/day [49]. Thus, "adequate" times were considered when the LPA and the number of steps were above 75thP, and sedentary time and the ST were below 25thP.

### Statistical analysis

Statistical analysis was performed with the Statistical Package for the Social Sciences (SPSS) program for Windows, version 20.0 (IBM Corporation®, New York, USA) and through the free statistical software R (R Development Core Team, 2014), version 3.2.2 ("Fire Safety"). The adopted level of significance was 5%. The Kolmogorov-Smirnov test and the asymmetry and kurtosis values showed non-normal data. Therefore, the results were presented as medians and interquartile intervals (IQR).

The LCA was performed in the poLCA (Polytomous Variable Latent Class Analysis) package [16] available in the R Statistical Software Library. The LCA is an analysis based on the subject’s characteristics. Thus, tests based on hypotheses are appropriate, procedures obtain the most adjusted, parsimonious, and interpretable model. This is a grouping method appropriate for identifying behavioral patterns [50] and analysis of iterations and associations between different types of variables related to the most common behaviors adopted by the pediatric population [51]. LCA was used to model the latent variable "movement behaviors" from five manifest variables related to adolescents PA and sedentary time.

The model’s quality was evaluated according to the relative and absolute adjustments values interpretation, degree of uncertainty and interpretability. The evaluation of the most parsimonious model was based on the Akaike Information Criterion (AIC), Bayesian Information Criterion (BIC), chi-square goodness adjustment test (its goodness- χ2) entropy (evaluation of the uncertainty degree), and maximum likelihood ratio test (G^2^). Finally, the probability of belonging to each item (ρ) and the classes prevalence (γ) allowed the homogeneity analysis and the model’s class separation. With this, it was possible to evaluate the models’ interpretability. All adjustment criteria selected were based on the LCA method proposed by Collins and Lanza [52].

The covariates groups related to individual, sociodemographic, family, and environmental correlates were associated with the movement behaviors classes. For this, a simple multinominal regression analysis was performed, with the regression coefficient (β), standard error (SE), odds ratio (OR) and 95% confidence interval (95% CI) values. Finally, a figure (S1 Fig) was presented with the beta regression coefficients (β) and 95% CI covariates values that were associated with the latent classes. Positive values (β+) indicated a higher chance of belonging to the less appropriate category of covariates to classes 2 or 3 than class 1. Negative values (β-) indicated a lower possibility of belonging to the less adequate category of covariates to classes 2 or 3 than class 1.

The Kruskal-Wallis test was used to verify the differences in the behavior variables’ quantitative values between the three latent classes that represented the movement behaviors. The Bonferroni post-hoc test was used to verify differences between pairs of groups. This correction was calculated by dividing the total significance value (α = 0.05) adopted by the number of comparations between the three latent classes. Thus, the value of the Bonferroni correction was equal to 0.0166. The effect’s Size was also calculated to complement and confirm the significance of the found associations, following Lenhard and Lenhard [53] recommendations. To this end, "Cohen’s d" values were calculated, and the values equal to or above 0.4 were considered desirable [54].

## Results

A total of 367 adolescents were evaluated, however, 58 were removed because they did not use the accelerometer properly. The sample consisted of 309 adolescents (15.37 ± 0.57 years), of whom 57% were female, and 70.9% belonged to the medium SS. 52.4% of the sample met the 60 minutes of MVPA recommendations, 75% spent less than 184.5 min/day in LPA (< 75thP) and 75% performed less than 10,107 steps/day (< 75thP). Regarding the sedentary time, only 25% of the sample was less than 10.8 h/day in total sedentary time (accelerometer) and less than 4h/day in ST (<25th P).

Model adjustment statistics for two to five classes were provided in Table 1. The model with three latent classes was chosen as the best model because it presented the best adjustment, entropy, and interpretability values compared to the others.

**Table 1:** Relative, absolute adjustment values, and adolescents LCA movement behavior models uncertainty degree.

	**AIC**	**BIC**	**DF**	**χ^2^**	**G^2^**	**p-G^2^**	**Entropy**
2 Classes	1712.47	1753.54	20	31.67	29.49	0.078	0.762
3 classes†	1705.71	1769.18	14	11.89	10.73	0.706	0.957
4 Classes	1711.06	1796.92	8	10.75	9.29	0.846	0.860
5 Classes	1720.37	1828.64	2	1.37	1.39	0.497	0.497

†Selected model with better adjustment values. Models with 6 classes or more presented negative degrees of freedom, so they were not presented.

AIC, Akaike Information Criterion; BIC, Bayesian Information Criterion; DF, degrees of freedom; χ^2^, Pearson’s chi-square test of goodness adjustment; G^2^, Likelihood Ratio; p-G^2^, Likelihood Ratio Test Statistics.

**Fig 1:**
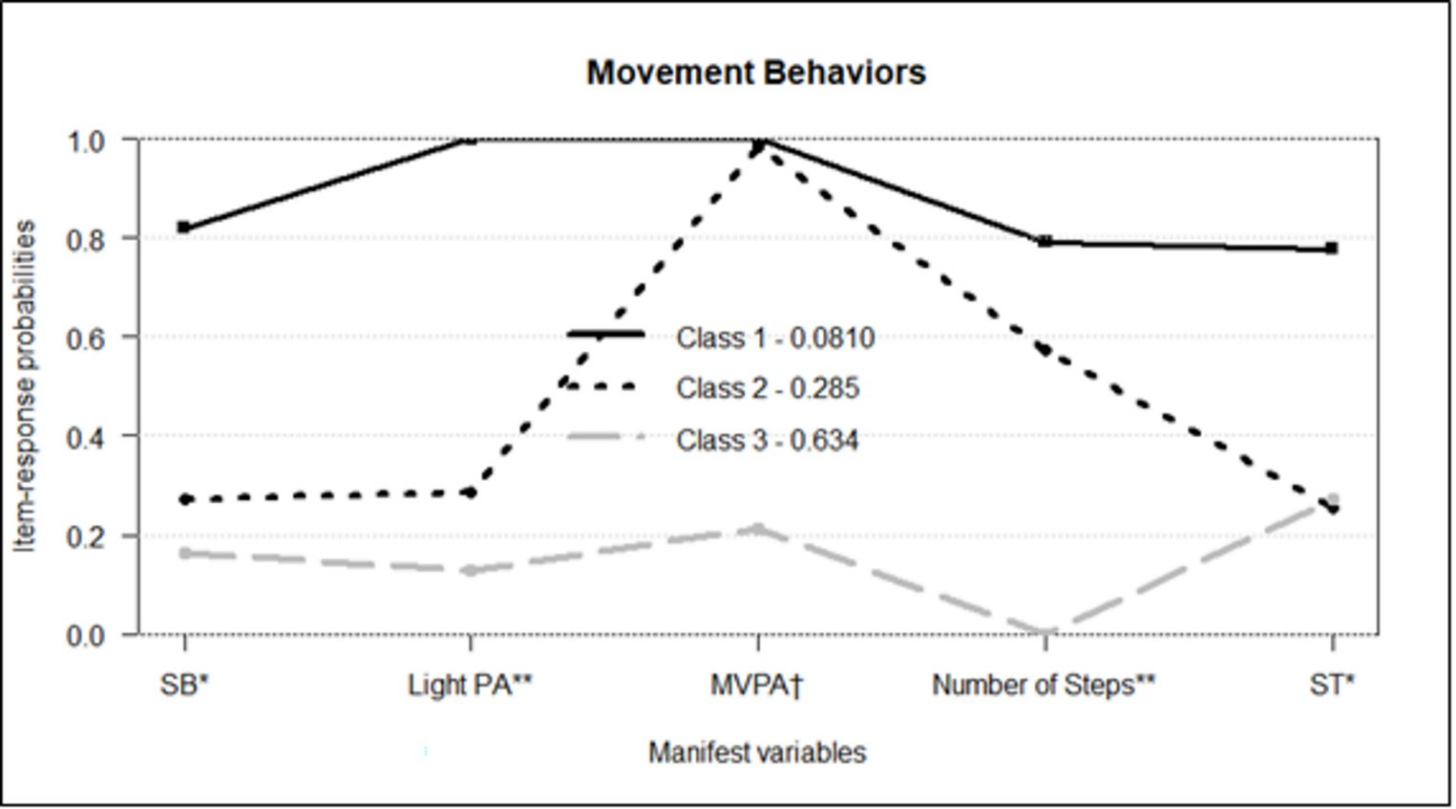
Adolescents’ LCA model of movement behaviors. Class 1: Active and Non-sedentary (γ: 8.10%); Class 2: Active and Sedentary (γ: 28.50%); Class 3: Inactive and Sedentary (γ: 63.40%). SB, sedentary time; PA, physical activity; MVPA, moderate to vigorous physical activity; ST, screen time. *Categorized by the 25^th^P percentile. ** Categorized by the 75t ^th^P percentile. †60-minute cut-off point.

The response probabilities to the three latent classes item (ρ) were presented in Fig 1, and labeled as: class 1, "Active and Non-Sedentary"; class 2, "Active and Sedentary" and class 3, "Inactive and Sedentary".

Class 1, "Active and Non-Sedentary", had a prevalence of 8.10%, and was considered the best class of movement behaviors. Adolescents in this class presented higher values of PA and less sedentary time when compared to the other classes. Class 2, "Active and Sedentary", had a 28.50% prevalence. Adolescents in this class presented moderate to high values for all expressions of PA, however, they had a high sedentary time when compared to the other classes. Finally, class 3, "Inactive and Sedentary", had the highest prevalence (63.40%); however, the adolescents in this class presented the lowest values of PA and high sedentary time when compared to the other classes.

Table 2 presented the associations of individual correlates (biological, lifestyle habits, and psychological) between latent classes and movement behaviors. No associations were found between classes 1 and 2 when individual covariates were analyzed. Between classes 1 and 3, it was observed that participants with low fruit intake, low CRF, and stressed, when compared to their peers (high fruit intake, high CRF and no stress), had respectively, 7.17 (95% CI: 1.64–31.43), 3.59 (95% CI: 1.14–11.26), and 3.56 (95% CI: 1.01–12.07) more chances to belong to class 3 (Inactive and Sedentary) than the best class (Active and Non-Sedentary).

**Table 2:** Association of individual covariates (biological, life habits, and psychological) with the adolescents’ movement behaviors LCA model.

	Class 1 / Class 2						Class 1 / Class 3					
Covariates	β	SE	OR	95%CI		p-value	β	SE	OR	95%CI		p-value
**Biological**												
Male^†^	1						1					
Female	-1.11	0.613	0.33	0.10	1.10	0.093	0.948	0.506	2.58	0.96	6.96	0.085
**Lifestyle Habits**												
No alcohol intake^†^	1											
Alcohol intake	0.468	0.605	1.60	0.49	5.23	0.454	0.913	0.527	2.49	0.89	7.00	0.109
High fruit intake ^†^	1											
Low fruit intake	1.269	0.685	3.56	0.93	13.62	0.089	1.97	0.754	7.17	1.64	31.43	0.022*
High vegetables intake^†^	1											
Low vegetable intake	-0.0002	0.6	1.00	0.31	3.24	1.00	-0.83	0.497	0.44	0.16	1.16	0.121
Low sugars intake^†^	1											
High sugars intake	-1.55	0.938	0.21	0.03	1.33	0.123	-0.422	0.34	0.66	0.34	1.28	0.239
Yes job^†^	1											
No job	-0.147	0.707	0.86	0.22	3.45	0.838	0.209	0.649	1.23	0.35	4.40	0.753
Active commuting^†^	1											
Passive commuting	0.193	0.533	1.21	0.43	3.45	0.723	0.352	0.606	1.42	0.43	4.66	0.572
High CRF (50^th^P) ^†^	1											
Low CRF (50^th^P)	0.081	0.644	1.08	0.31	3.83	0.902	1.277	0.584	3.59	1.14	11.26	0.049*
**Psychological**												
No stress^†^	1											
Stressed	1.25	0.633	3.49	1.01	12.07	0.071	1.27	0.561	3.56	1.01	12.07	0.042*
Never lonely^†^	1											
Loneliness	-0.527	0.674	0.59	0.16	2.21	0.449	-0.113	0.59	0.89	0.28	2.84	0.851
No sadness^†^	1											
Yes sadness	-1.21	0.625	0.30	0.09	1.02	0.076	0.09	0.512	1.09	0.40	2.98	0.859

*Significative association.

^†^Reference category.

β, Multinominal regression coefficient; SE, Standard error; OR, Odds Ratio; 95%CI, 95% Confidence interval. -, values not found; SS, socioeconomic status; CRF, cardiorespiratory physical fitness.

The associations of sociodemographic, family, and environmental covariates between the movement behaviors classes were presented in Table 3. Regarding sociodemographic variables, positive associations were verified for the head of the family’s schooling, and negative associations were verified for the SS among the behavior classes. Adolescents whose head of the family obtained an “elementary school” level education, as opposed to “higher education” had 4.40 (95% CI: 1.23–15.80) more chances of belonging to the "Inactive and Sedentary" class than the "Active and No sedentary" class. For the SS, it was observed that in comparison to adolescents classified with low SS, those categorized with medium and high SS had 82% (OR: 0.18, 95% CI: 0.04–0.77) and 83% (OR: 0.17, 95% CI: 0.04–0.65) less chances to belong to the "Active and Non-sedentary" class when compared to the "Active and Sedentary" and "Inactive and Sedentary" classes, respectively. No associations were found between behavior classes when family covariate was analyzed.

**Table 3:** Sociodemographic, family, and environmental covariates association with adolescent’s movement behaviors LCA model.

	Class 1 / Class 2						Class 1 / Class 3					
Covariates	β	SE	OR	95%CI		p-value	β	SE	OR	95%CI		p-value
**Sociodemographic Correlates**												
High and Medium ss^†^	1											
Low ss	-1.69	0.73	0.18	0.04	0.77	0.039*	-1.78	0.685	0.17	0.04	0.65	0.023*
Higher Education^†^	1						1					
High school	0.114	0.402	1.12	0.51	2.46	0.783	-0.33	0.709	0.72	0.18	2.89	0.963
Elementary school	0.864	0.454	2.37	0.97	5.78	0.094	1.482	0.652	4.40	1.23	15.80	0.05*
Never studied	0.979	1.111	2.66	0.30	23.49	0.404	2.192	1.243	8.95	0.78	102.34	0.116
**Family Correlates**												
Active MVPA^†^	1						1					
Insufficient Active	1.11	0.909	4.10	0.69	24.35	0.147	1.185	0.854	3.27	0.61	17.44	0.051
**Environmental Correlates**												
Best Land use mix-diversity^†^	1						1					
Worst Land use mix-diversity	-3.21	1.814	0.04	0.00	1.41	0.102	-2.56	1.8	0.08	0.00	2.63	0.179
Best residential density^†^	1						1					
Worst residential density	-13.4	0.396	0.00	0.00	-	-	-12.59	0.306	0.00	0.00	0.00	-
Best land use mix-access^†^	1						1					
Worst land use mix-access	-1.804	0.643	0.16	0.05	0.58	0.016*	-0.887	0.557	0.41	0.14	1.23	0.137
Best street connectivity^†^	1						1					
Worst street connectivity	-1.87	0.76	0.15	0.03	0.68	0.03*	-1.465	0.703	0.23	0.06	0.92	0.059
Walking/cycling ease^†^	1						1					
No Walking/Cycling ease	-1.73	0.605	0.18	0.05	0.58	0.014*	-1.167	0.57	0.31	0.10	0.95	0.063
Pedestrian & automobile traffic safety^†^	1						1					
No pedestrian & automobile traffic safety	-1.69	0.655	0.18	0.05	0.67	0.024*	-0.777	0.559	0.46	0.15	1.38	0.192
Crime Safe^†^	1						1					
Not-Crime Safe	1.384	0.814	3.99	0.81	19.68	0.115	0.417	0.529	1.52	0.54	4.28	0.444
Best neighborhood aesthetics^†^	1						1					
Worst Neighborhood aesthetics	0.742	0.634	2.10	0.61	7.28	0.265	0.845	0.573	2.33	0.76	7.16	0.166

*Significative association.

^†^Reference category.

β, Multinominal regression coefficient; SE, standard error; OR, Odds Ratio; 95%CI, 95% Confidence interval; -, values not found; MVPA, moderate to vigorous physical activity.

In relation to environmental covariates, compared to adolescents living in neighborhoods with "worse access to diversified land use", "worst street connectivity", "worst walking/pedaling ease" and "worst traffic safety"; those living in neighborhoods with "better access to diverse land use", "better street connectivity", "better walking/pedaling ease" and "better traffic safety" had respectively 84% (OR: 0.16, 95% CI: 0.05–0.58), 85% (OR: 0.15, 95% CI: 0.03–0.68), 82% (OR: 0.18, 95% CI: 0.05–0.58) and 82% (OR: 0.18, 95% CI: 0.05–0.67) less chances of belonging to the "Active and Non-Sedentary" class than to the "Active and Sedentary" class.

The variables in Tables 2 and 3 that had a significant statistically association between the classes of behavior can be seen in S1 Fig.

Table 4 exposes the quantitative variables values variability evaluation amongst the three latent classes. For the individual correlates, a higher fruits intake was found in the "Active and Non-Sedentary" class than class 2 and class 3. Regarding CRF, higher values were found in class 2 than in class 3.

**Table 4:** Continuous variables variability related to individual, family, and environmental covariates.

	Class 1: Active and Non-Sedentary		Class 2: Active and Sedentary		Class 3: Inactive and Sedentary			
Quantitative covariables	Median	25thP - 75thP	Median	25thP - 75thP	Median	25thP - 75thP	*p*	ES (d-Cohen)
**Individual Correlates**								
Age (years old)	16	15–16	15	15–16	15	15–16	0.214	-
CRF (scores)	33	29.7–35	31.7^c^	29.4–36.2	30.2^c^	28.4–33.2	0.002*	0.377
Fruit intake (Number/week)	5^a^^,^^b^	3.0–6	3^a^	2.0–5	3^b^	2.0–5	0.009*	0.315
Vegetables intake (Number/week)	3	2.5–5.5	4	3–6.7	4	3.0–6	0.451	-
Sugar intake (Number/week)	2	1.0–3	3	1.2–4.7	2	1.0–4	0.295	-
**Environmental Correlates**								
Land use mix-diversity	126^a^	112–145	142.5^a^	126.5–161	143	121–163.5	0.03*	0.259
Residential density	1.8^a^^,^^b^	1.6–2	2.1^a^^,^^c^	1.8–2.5	2.4^b^^,^^c^	2–2.6	<0.001*	0.678†
Land use mix-diversity-access	2.5^b^	2.1–2.8	2.8	2.3–3	2.8^b^	2.5–3	0.018*	0.283
Street connectivity	3	2.3–3.6	2.6	2.3–3.3	2.6	2.3–3.3	0.27	-
Walking/Cycling facilities	2.6	2.0–3	2.6	2.3–3	2.6	2.3–3	0.509	-
Traffic safety	2.5	2.3–2.7	2.3^c^	2.1–2,7	2.6^c^	2.3–3	0.021*	0.277
Crime Safety	1.8	1.4–2.4	1.7	1.4–2.4	2	1.4–2.3	0.909	-
Neighborhood aesthetics	1.7^a^^,^^b^	1.3–2.2	2.3^a^	2–2.7	2.2^b^	1.7–2.7	0.005*	0.344

*Significant difference between the 3 groups using the Kruskal-Wallis test (p <0.05).

^a^Significant difference according to the Bonferroni post-hoc test (p≤0.016) between class 1 and class 2.

^b^Significant difference according to the Bonferroni post-hoc test (p≤0.016) between class 1 and class 3.

^c^Significant difference according to the Bonferroni post-hoc test (p≤0.016) between class 2 and class 3.

†Effect Size (d-Cohen) Desired ≥0,400.

ES, effect size; ACC, accelerometer; CRF, cardiorespiratory physical fitness.

With regard to the built environment factors, it was observed that the adolescents who are in the "Active and Non-Sedentary" class, were those who live in neighborhoods with the lowest values for neighborhood attributes—"Diversified land use", "Residential density", "Access to diversified land use", "Pedestrian and car traffic safety" and "Neighborhood Aesthetics"; when compared to adolescents belonging to class 2 (Active and Sedentary) and / or 3 (Inactive and Sedentary). Table 4 refinement with Bonferroni’s post-hoc specific significance values, and their respective effect sizes, can be found in S1 Table. It is important to note that the significant variations and differences observed showed a desirable effect size (≥0.4).

## Discussion

This study identified movement behaviors latent classes of Brazilian adolescents based on five manifest variables related to PA (LPA, MVPA, number of steps) and sedentary time (total sedentary time and ST). Our results contributed to a better understanding of adolescents’ behavioral patterns and their association with a variety of correlates (individual, sociodemographic, family, and environmental). To date, a small variety of correlates have been explored in studies dedicated to analyzing the groupings of behaviors related to activity and health.

Consistent patterns of lifestyle-related behaviors were identified in previous studies only using behavior models related to PA and sedentary time [12, 13, 17] and models using different health behaviors, in addition to PA and sedentary time, such as BMI, eating habits and sleep duration, alcohol and tobacco intake [18, 55, 56]. In addition, out of these studies, only Parker et al. [13] associated activity related behaviors classes with a set of correlates–intrapersonal, interpersonal and physical.

The best-adjusted model was the one with three latent classes. None of the classes identified encompassed inactive and non-sedentary adolescents. Class 1, "Active and Non-Sedentary", despite having the lowest prevalence (8.10%), was the healthiest class in terms of PA and sedentary time. Class 2, "Active and Sedentary" comprised 28.5% of the sample, although 100% met the recommendations of MVPA, only 25% met the sedentary time cut-off points. Class 3, "Inactive and Sedentary" was the largest group (63.4%), however was the least healthy class in terms of PA and sedentary time. The finding that the best behavior class comprised the lowest proportion of adolescents is consistent with previous research [13, 17, 18] and is something concerning. At the same time, there is a higher prevalence of adolescents with lower PA values and more sedentary time. However, the differences may in part reflect differences related to the SS of the sample, since the adolescents in the best class were also the ones with the lowest SS. Thus, the greater engagement in PA could also be explained by economic needs, which often make these adolescents have to help with chores at home or need to have a formal job. Future research focusing on individuals with the best behavioral pattern is important, so that we can learn the best way to intervene and improve the inappropriate behaviors of the majority of adolescents.

Models of latent classes with characteristics resembling those of the present study, where unhealthy and healthy behaviors overlap in adolescents, such as "Active and Non-Sedentary", "Inactive and Sedentary" were observed in previous studies [12, 17, 18, 57, 58]. A study that deserves special attention was developed by Faria et al. [17] and involved Brazilian adolescents, aged between 15 and 18 years, and the same variables as the present study were included in the LCA model. The variables were grouped into three classes, two of which were also demonstrated here (Active and Non-Sedentary and Inactive and Sedentary). Together, the findings of the present study and Faria et al. [17] show two similar behavioral patterns in Brazilian adolescents, a healthy pattern and other unhealthy one. Understanding the factors that make these individuals stand out in the highest risk group (“Inactive and Sedentary”) can provide valuable guidance for the development of successful interventions, so that they can modify their behavior, moving to the lowest risk group.

Also, it was possible to test the influence of the correlates among the behavior’s classes. There were no associations between the behavior classes with the family correlate, which demonstrates that the head of the family’s PA level did not interfere with the behavior classes of the present sample. Although the role of parents and family is important, they represent only one of the many agents of socialization of adolescents [59]. It is possible that during adolescence the participation of peers tends to be more significant than that of parents/legal guardians, a factor that was not controlled in the present study, but observed in previous studies, such as the support of siblings and friends, which may favor the participation of adolescents in PA [60, 61]. Future research should be carried out with other family-related characteristics that were not investigated here.

Among the examined individual correlates, there were associations between the best (Active and Non-Sedentary) and the worst behaviors class (Inactive and Sedentary) only for the variables related to lifestyle habits (fruit intake and CRF) and to psychological (stress). Class 3 was characterized by presenting the unhealthiest behaviors—low fruit intake, low CRF and stress level. It is possible that the characteristics from this class such as lower PA and higher sedentary time contributed to a low CRF and to stress level. Similarly, by identifying risk behaviors classes, including physical fitness variables, PA, sedentary time, eating habits and body composition, Tabacchi et al. [62] observed that adolescents in the "Low PA /sport" class were also those at higher risk of unhealthy behaviors, such as obesity, high sedentary time, and low CRF values when compared to the healthiest class (high PA, low sedentary time, adequate eating habits, high CRF, and low depression risk). These results align with ours and show that inappropriate behaviors can cluster with other important variables, or behaviors related to health. Therefore, more research is needed to establish consistency in the findings and, if necessary, create strategies to modify these inappropriate behaviors.

Adolescents in the worst class presented higher stress levels. Thus, a closer look at this result is needed, as high stress levels can contribute to future mental health problems such as anxiety and depression. Faria et al. [17] reported that adolescents in the "Inactive and Sedentary" class presented higher final scores for common mental disorders related to anxiety and depression symptoms compared to those in the "Active and Non-sedentary" class. Both results support previous studies that demonstrated that inadequate PA levels, few hours of sleep, and high sedentary time, such as watching TV and computer use, were associated with poor mental health in young people [63, 64]. All these findings demonstrate the need for a better understanding of the factors that may be triggering psychological impairments in the young population, so that specific interventions can be executed.

Adolescents whose head of the family had "elementary school" when compared to those with "higher education" were more prone to belonging to the low PA and high sedentary time class than the healthiest class. Tabacchi et al. [62] also found that adolescents belonging to the class characterized by "low PA and high sedentary time" had parents with low level of education. These results indicate the parents with higher education tend to have greater knowledge of health-related aspects, and in turn, encourage adolescents to participate in more PA and reduce sedentary time. Thus, to purpose public policies that improve the educational level of parents can, indirectly, contribute to improving adolescents’ health.

One of the present study’s remarkable findings was that adolescents identified with low SS were more likely to belong to the "Active and Non-Sedentary" class than to the other "Active and Sedentary" and "Inactive and Sedentary" classes, when compared to those of middle and high SS. The results found do not support the premise that individuals with a medium and high SS usually engage in more PA [15, 65] and spend less time in sedentary activities [21]. This result may be associated with the participants’ economic needs, that encourage them to perform domestic chores, such as washing dishes, sweeping the house, taking care of the younger siblings, among other daily tasks, which contributes to a greater PA engagement, especially LPA, which had a higher time proportion in class 1 compared to the others. The contribution of domestic activities in children and adolescents’ daily PA amount has been demonstrated in other studies [61, 66].

Regarding environmental correlates, the adolescents who perceived their neighborhoods with "worse diversified land use, worse street connectivity, worse walk/pedal ease and without safety in car and pedestrian traffic" had more chances to belong to behavior’s best class "Active and Non-Sedentary" than to the "Active and Sedentary" class. Although some studies demonstrate that favorable physical environments, such as higher residential density, better diversified land use, with cobbled and connected streets, with greater security against crime, near parks and commerce [67–70] are associated with longer PA time and lower sedentary time, this was not observed in the present study.

While it is difficult to make direct comparisons because few behavior groups studies evaluate these environmental factors, we only found a single study with latent classes [13], however, with different results from this study. By associating the behaviors classes with the correlates "safety against crime" and "safety in car and pedestrian traffic", Parker et al. [13] observed that the perception of greater safety traffic was associated with a greater chance of belonging to the “Highly Active, Low Sedentary” class rather than to the “Physically Inactive, Highly Sedentary” class. Such differences can be partially explained by the characteristic’s diversity among the studies cities, such as the size, demographic density, and number of inhabitants. It is also important to recognize the differences between these two countries in terms of crime and traffic safety, which may reflect on the different adolescents’ behavior patterns. While in Brazil, for every 100,000 inhabitants, an average of 30 homicides and 16 traffic deaths occur, in Australia these values are low and correspond, on average, to less than 1 homicide and less than 5 traffic deaths [71, 72].

It is important to highlight that individuals from both classes (1 and 2) met the MVPA recommendations, what differentiates them is that young people from environments perceived as unfavorable had a longer time in LPA and lower sedentary time. One coincidence in the present study results was that both adolescents who perceived the environment with worse attributes and those with low SS had a higher chance of belonging to the "Active and Non-sedentary" class compared to the "Active and Sedentary" class. It is speculated that the SS has an important influence on this relationship, for example, a lower family income may be associated with fewer TVs at home, the absence of computers and video games, which in turn may favor the less time spent in sedentary activities. It may also be associated with individuals who live in neighborhoods distant from the center and / or the schools, and who need to move on foot or through public transport, which may potentiate LPA. Future research should further explore the factors that can influence in the LPA and the sedentary time.

When advancing the analysis, it was possible to verify the variability of the quantitative variables between classes (Table 4 and S1 Table). A consumption of two more fruits per week was observed in class 1 in comparison to the other classes was observed, which may be associated with the best behavioral habits of this group, as already observed in a previous study [73]. In addition, class 2 presented higher CRF values than class 3. The fact that class 2 adolescents comply with the MVPA recommendations, different from those in the "Inactive and Sedentary" group, can explain the better CRF results. This fact was also demonstrated by Morrow et al. [74], where adolescents who did not comply with the aerobic activities and muscle strengthening recommendations were more likely not to reach satisfactory CRF levels. These differences between behavioral of classes underscore the need to encourage adolescents to have better habits and behaviors, such as consuming more fruits, performing more PA and better CRP levels.

Regarding the environment factors, it was observed that the adolescents from the healthiest class are those who live in neighborhoods with the lowest values for attributes–"Land diversity use", "Residential density", "Access to diversified land use", "Pedestrian and car traffic safety" and "Neighborhood Aesthetics" when compared to adolescents to the "Active and Sedentary" and / or "Inactive and Sedentary" classes. As previously stated, it is observed that the more inactive (class 3) and sedentary (classes 2 and 3) the adolescents were, the better values were attributed to the neighborhood environment, which allows us to conclude that the more unfavorable physical environments were potentiating more PA and less sedentary time in the present sample. Despite being the first study in small countryside city, without adequate urban planning, these findings should be examined with caution. It is essential to highlight that a recent study conducted by our group [47], analyzing latent classes of built environment and its association with PA and sedentary time, demonstrated consistent results. The individuals who lived in more unfavorable neighborhoods performed about 20 minutes more LPA per day and performed less sedentary activities compared to residents of the best neighborhood. Further research is required to determine whether there are other additional aspects of the neighbourhood that may also influence class membership.

This study has some limitations that should be mentioned. First, we could not determine the associations’ causality due to the study’s cross-sectional design. Second, the dichotomization of variables may have led to the attenuation of some results, however this is usually done to facilitate the understanding of the information derived from the LCA analyses. Third, due to the lack of validated cut-off points for the classification of some manifest and covariate variables, cut-off points were adopted based on the sample percentiles (25 and 75). It is worth emphasizing that the 25th and 75th percentiles were applied in other LCA studies [17, 18, 47] and can be useful for comparing adolescents and their peers. Finally, the subjectivity associated with the questionnaires used to collect information from the correlates, although this is the only way to collect some of this information.

The use of LCA to identify movement behaviors classes using a combination of objective and subjective measures can be highlighted as this study’s strength, especially because it is an approach still little explored in Brazilian studies. In addition, it should be considered that several expressions of PA (LPA, MVPA, and number of steps) and different sedentary time (sedentary time and ST) were analyzed employing objective and subjective measures. Another important consideration was the inclusion of various potential correlates groups (individual, family, sociodemographic and environmental) associated with movement behaviors, different from most studies, which generally use only one or two correlates groups. In addition, this study adds important information about possibilities of the neighborhood environment correlates that may be associated with the classes of movement behaviors identified and should receive greater attention, mainly in developing countries such as Brazil, where there are few studies that investigate these environmental characteristics in the behaviors of adolescents.

Although plentiful literature identifies ecological correlations of participation in individual behaviors related to the activity (e.g., PA or sedentary time), understanding potential influences on combinations of these behaviors among adolescents is limited. A possible explanation for this may be associated with the different methods used in the studies that make it difficult to compare this information between different demographic groups. Thus, the use of more consistent methods in the studies will allow direct comparisons between various demographic groups. Thus, future research should identify other potential correlates that may be related to groups of behaviors associated with PA and sedentary time, and therefore assist in the promotion of strategies of appropriate multidimensional interventions to promote PA, reduce sedentary time and enable healthier lifestyle habits.

## Conclusion

A three latent classes model was originated from the analysis of five manifest variables (LPA, MVPA, number of steps, sedentary time and ST), representing the behaviors patterns of our sample. Associations were identified between the three latent classes of behaviors for individual, sociodemographic and environmental correlates, but this was not evidenced for the family correlate.

Adolescents living in neighborhoods with the highest values for the neighborhood attributes (use of soil diversity, residential density, access to diversified land use, safety of pedestrian and car traffic and neighborhood aesthetics) had a lower probability of belonging to the "Active and Non-Sedentary" class when compared to the "Active and Sedentary" class. Those with medium and high SS were less likely to belong to class 1 than classes 2 and 3, respectively. In addition, adolescents with low fruit intake, low-grade CRF and adolescents whose head of the family obtained an “elementary school” level education were more likely to belong to the class "Inactive and Sedentary" when compared to those in the "Active and Non-Sedentary" class. The low prevalence of individuals in the "Active and Non-Sedentary" class than in the other two less active and more sedentary classes emphasizes the need for intervention. Identifying a set of sociodemographic and environment factors associated with the best class (Active and Non-Sedentary) can help identify ways to help other adolescents achieve an optimal mix of movement behaviors.

Thus, identifying the primary individual, sociodemographic, family, and environmental correlates associated with different classes of movement behaviors is essential to obtain insights on effective behavioral change strategies for those who are most at risk of engaging in unhealthy behavioral classes.

## Supporting information

S1 Table Significance values (p) of the Bonferroni post-hoc test and their respective Effect Size (ES) values (d-Cohen). (DOCX)

S1 Fig Regression coefficient (β) values and the 95% confidence intervals (95% CI) of the association between the covariates with the latent classes that represent the adolescents’ movement behaviors. Graph A: Class 1, Active and Non-Sedentary versus Class 2, Active and Sedentary; Graph B: Class 1, Active and Non-Sedentary versus Class 3, Inactive and Sedentary. SS, socioeconomic status; CRF‡, cardiorespiratory physical fitness (50 ^th^P percentile); #head of household’ education level.
(TIF)
