# Supplementary material for: Ecological correlates related to adolescent movement behaviors: A latent class analysis
Source: PLoS One. 2022 Jul 21;17(7):e0271111. doi: 10.1371/journal.pone.0271111 (PMC9302818; doi:10.1371/journal.pone.0271111)
Supplement: S1 Table — (DOCX) [file pone.0271111.s001.docx]

**Supplementary Table 1. Significance values (p) of the Bonferroni post-hoc test and their respective Effect Size (ES) values (d-Cohen).**

| **Quantitative covariables** | **C1 *versu*s** **C2(a)** | **ES**  **(d-Cohen)**  **(a)** | **C1 *versus*** **C3(b)** | **ES**  **(d-Cohen) (b)** | **C2 *versus*** **C3(c)** | **Es**  **(d-Cohen) (c)** |
| --- | --- | --- | --- | --- | --- | --- |
| **Individual Correlates** | | | | | | |
| Age (years old) | - | - | - | - | - | - |
| CRF (scores) | 0,887 | - | 0,027 | - | 0,002** | 0,379 |
| Fruit intake (Number per week) | 0,005** | 0,545† | 0,016** | 0,32 | 0,102 | - |
| Vegetables intake (Number per week) | - | - | - | - | - | - |
| Sugar intake (Number per week) | - | - | - | - | - | - |
| **Environmental Correlates** | | | | | | |
| Land use mix-diversity | 0,006** | 0,538† | 0,018 | 0,323 | 0,619 | - |
| Residential density | 0,001** | 0,638† | <0,001** | 0,72† | <0,001** | 0,428† |
| Land use mix-diversity-access | 0,034 | - | 0,004 | 0,224 | - | - |
| Street connectivity | - | - | - | - | - | - |
| Walking/Cycling facilities | - | - | - | - | - | - |
| Traffic safety | 0,364 | - | 0,39 | - | 0,006** | 0,329 |
| Crime safety | - | - | - | - | - | - |
| Neighborhood aesthetics | 0,002** | 0,607† | 0,003** | 0,411 | 0,362 | - |

*Significant difference between the three groups using the Kruskal-Wallis test (p <0.05).

(a) Significant difference according to the Bonferroni post-hoc test (p≤0.016) between class 1 versus class 2.

(b) Significant difference according to the Bonferroni post-hoc test (p≤0.016) between class 1 versus class 3.

(c) Significant difference according to the Bonferroni post-hoc test (p≤0.016) between class 2 versus class 3.

†Effect Size (Effect Size - d-Cohen) Desired ≥0,400; - In the significant association.

Class 1: Active and Non-Sedentary MBAL; Class 2: Active and Sedentary MBAL; Class 3: Inactive and Sedentary MBAL; ES: Effect size; ACC: Accelerometer; CRF: Cardiorespiratory physical fitness.
