# Supplementary figures and images for: Ecological correlates related to adolescent movement behaviors: A latent class analysis
Source: PLoS One. 2022 Jul 21;17(7):e0271111. doi: 10.1371/journal.pone.0271111 (PMC9302818; doi:10.1371/journal.pone.0271111)

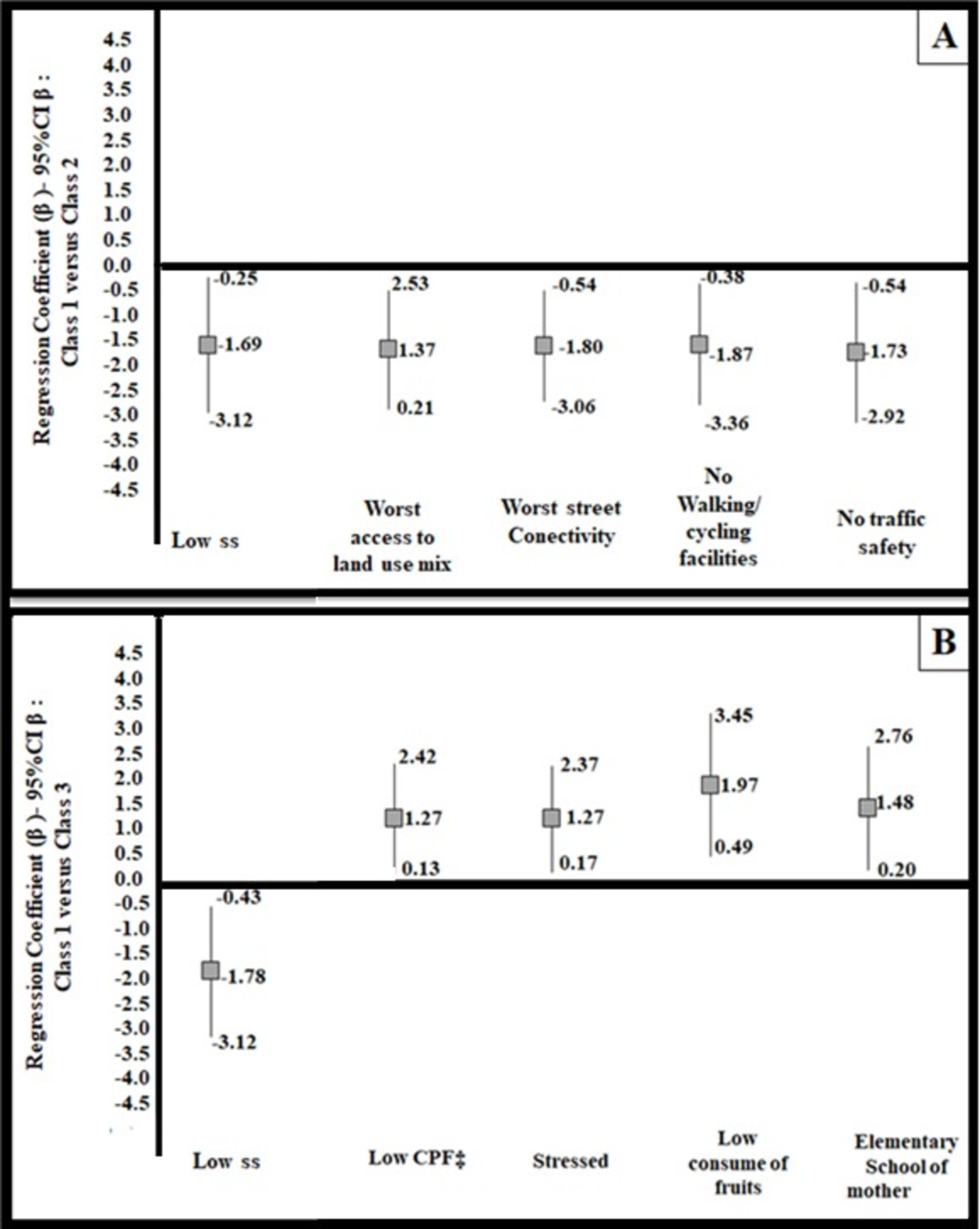

Supplement: S1 Fig — Graph A: Class 1, Active and Non-Sedentary versus Class 2, Active and Sedentary; Graph B: Class 1, Active and Non-Sedentary versus Class 3, Inactive and Sedentary. SS, socioeconomic status; CRF‡, cardiorespiratory physical fitness (50 thP percentile); #head of household’ education level. (TIF) [file pone.0271111.s002.tif]
